# Impact of Definitive Therapy with Beta-Lactam Monotherapy or Combination with an Aminoglycoside or a Quinolone for *Pseudomonas aeruginosa* Bacteremia

**DOI:** 10.1371/journal.pone.0026470

**Published:** 2011-10-26

**Authors:** Ioannis A. Bliziotis, Nicola Petrosillo, Argyris Michalopoulos, George Samonis, Matthew E. Falagas

**Affiliations:** 1 Alfa Institute of Biomedical Sciences (AIBS), Athens, Greece; 2 National Institute for Infectious Diseases “L. Spallanzani”, Rome, Italy; 3 Intensive Care Unit, “Henry Dunant” Hospital, Athens, Greece; 4 Department of Internal Medicine, University Hospital of Heraklion, Heraklion, Greece; 5 Department of Medicine, “Henry Dunant” Hospital, Athens, Greece; 6 Department of Medicine, Tufts University School of Medicine, Boston, Massachusetts, United States of America; Los Angeles Biomedical Research Institute, United States of America

## Abstract

**Background:**

Bacteremia by *Pseudomonas aeruginosa* represents one severe infection. It is not clear whether beta-lactam monotherapy leads to similar rates of treatment success compared to combinations of beta-lactams with aminoglycosides or quinolones.

**Methods:**

Retrospective cohort study from 3 tertiary hospitals (2 in Greece and 1 in Italy). *Pseudomonas aeruginosa* isolates were susceptible to a beta-lactam *and* an aminoglycoside *or* a quinolone. Patients received appropriate therapy for at least 48 hours. Primary outcome of interest was treatment success in patients with *definitive* beta-lactam combination therapy compared to monotherapy. Secondary outcomes were treatment success keeping the same empirical and definitive regimen, mortality, and toxicity.

**Results:**

Out of 92 bacteremias there were 54 evaluable episodes for the primary outcome (20 received monotherapy). Treatment success was higher with combination therapy (85%) compared to beta-lactam monotherapy (65%), however not statistically significantly [Odds ratio (OR) 3.1; 95% Confidence Interval (CI) 0.69–14.7, p = 0.1]. Very long (>2 months) hospitalisation before bacteremia was the only factor independently associated with treatment success (OR 0.73; 95% CI 0.01–0.95, p = 0.046), however this result entailed few episodes. All-cause mortality did not differ significantly between combination therapy [6/31 (19%)] and monotherapy [8/19 (42%)], p = 0.11. Only Charlson comorbidity index was associated with excess mortality (p = 0.03).

**Conclusion:**

Our study, in accordance with previous ones, indicates that the choice between monotherapy and combination therapy may not affect treatment success significantly. However, our study does not have statistical power to identify small or moderate differences. A large randomized controlled trial evaluating this issue is justified.

## Introduction

Bacteremia caused by *Pseudomonas aeruginosa* represents one of the most severe infections in the hospital setting, especially for immunocompromised or critically ill patients. Recent studies report overall mortality in patients with this infection between 20% and 60% and mortality solely attributed to the infection around 15% [1], [2], [3], [4], [5]. An important factor, which complicates these infections, is multidrug-resistance that is very common among *Pseudomonas aeruginosa* strains globally, leaving a small number of appropriate antimicrobials, which are *in vitro*, at least, active against them [4], [6], [7]. Consequently, a number of studies have highlighted the possible negative effect of inappropriate empirical antimicrobial therapy on length of hospital stay and other unwanted outcomes [8]. In addition, there are reports indicating that early initiation of appropriate therapy may also play significant role regarding response to treatment [9], [10].

The use of antibiotic combinations represents a common therapeutic approach against infections with *Pseudomonas aeruginosa*, used for decades [11], [12], [13], [14]. The rationale for using antibiotic combinations lies on their different mechanisms of action, their *in vitro* synergy and the wide antimicrobial spectrum that they offer [6], [7], [14]. However, the administration of more than one antimicrobial increases toxicity as well as possible drug interactions between the antimicrobials and with other medications [15], [16]. In addition, there is further increase in antimicrobial resistance by increased antimicrobial use [17], [18], [19], [20]. During the last 15 years several original papers and systematic reviews questioned the benefit of using beta-lactams in combination with other antimicrobials for various serious bacterial infections in immunocompetent and immunocompromised patients [15], [16], [21], [22], [23].

Despite the fact that the benefit from combinations of beta-lactams with other antimicrobials has been considered equivalent to beta-lactam monotherapy for many serious infections, it is not clear whether this is true for *Pseudomonas aeruginosa* bacteremia [6], [22], [23]. Few papers focused exclusively in this issue, especially regarding definitive therapy, based on susceptibility testing results [24], [25], [26]. In this retrospective study we review *Pseudomonas aeruginosa* bacteremias in which beta-lactam monotherapy and combination therapy with an aminoglycoside or a quinolone were both therapeutic options according to antimicrobial susceptibility test results. We will assess how the two different therapeutic choices of the clinicians affected clinical success and other outcomes.

## Methods

### Setting, study population

This retrospective cohort study included patients from two tertiary hospitals in Greece and one in Italy. Specifically, we used records of patients from “Henry Dunant” Hospital in Athens, University Hospital of Heraklion in Crete, and from the National Institute for Infectious Diseases “L. Spallanzani” in Rome. The study period differed in the three centers mainly due to variability in the available data included in the medical records of patients throughout the study. Thus, in “Henry Dunant” hospital patient records were reviewed from January 2002 until August 2005, in Italy from January 2001 until August 2005, whereas in Heraklion from February 2003 until August 2007. However, data collection took place at the same time in the three centres using a specific case report form.

### Inclusion criteria

First, patients with bacteremia from *Pseudomonas aeruginosa* were identified by the electronic databases of the microbiology laboratories of the hospitals. In some cases the relevant antibiograms were readily available, whereas in others data had to be found in patients' hospital records. The *Pseudomonas aeruginosa* isolates should be susceptible to a beta-lactam *and* an aminoglycoside *or* a quinolone (mainly ciprofloxacin) so that comparisons between patients that received beta-lactam monotherapies and combination therapies would be meaningful. Another inclusion criterion was the administration of at least one active beta-lactam, *with* or without an aminoglycoside, or quinolone (according to the antimicrobial susceptibility testing results) for at least 48 hours. Patients with highly resistant polymyxin-only-susceptible strains, which were rather common during this period, were excluded from further analysis. Moreover, all patients should have symptoms indicative of infection, based primarily on the definitions of systemic inflammatory response syndrome [27].

Primary bacteremias and catheter-related bloodstream infections were diagnosed according to definitions Infectious Diseases Society of America [28]. Multiple episodes of bacteremia in the same patient were included in the analysis as long as they occurred more than three weeks apart and the patient did not have any symptoms, signs, or findings consistent with an infection for at least a week between the two episodes. Only adult patients were included in the study.

### Data collection

Various baseline characteristics of the patients at the time of the bacteremia that could affect the outcome of the infection were collected, recorded and analyzed. Variables included age, gender, and comorbidities of patients (including solid tumors, hematologic malignancies, neutropenia, recent chemotherapy, HIV infection, acute and chronic renal failure, respiratory insufficiency, or cardiac disorders), duration of hospitalization before the occurrence of bacteremia, as well as surgeries, ICU hospitalization, and existence of central venous catheter prior to the bacteremia. In addition, the site of initial infection was recorded in secondary bacteremias.

Charlson comorbidity index (CCI) was calculated for all patients [29], [30]. This index takes into account 19 conditions and categories of diseases, including congestive heart failure, dementia, tumors, metastatic disease, AIDS, different degrees of hepatic disease, moderate to severe renal disease, diabetes, connective tissue disease and hematological malignancies, to name a few. A large number of conditions that have been encoded in ICD-9 have received specific grading according to CCI, which we utilized [31].

### Antimicrobial therapy

Both patients that received empirical and definitive antimicrobial therapies against *Pseudomonas aeruginosa* bacteremia were evaluated. Therapy was considered empirical if it was initiated no later than 24 hours after the specimen from which the *Pseudomonas aeruginosa* strain was isolated and definitive if it was initiated or continued after the result of the blood culture and the relevant susceptibility testing was available to the clinicians. When a patient received appropriate empirical therapy that was not changed after the susceptibility testing result was available, patient was evaluated twice, both regarding the empirical and definitive therapy. Only the first definitive (i.e. based on susceptibility testing) treatment administered to a patient during an episode of bacteremia was evaluated for the outcomes of interest and not any further changes. Monotherapy was considered any therapy that included only a beta-lactam antibiotic. Combination therapy included a beta-lactam antibiotic plus either an aminoglycoside or a quinolone. Patients that had received three or more active antibiotics (e.g. an aminoglycoside plus ciprofloxacin plus a beta-lactam) were excluded from the analysis. Similarly, no other regimen that included agents active against *Pseudomonas aeruginosa* (e.g. colistin) was evaluable. All patients included in the study received commonly used doses of antibiotics for sufficient time, in concordance with various guidelines [28], [32], [33], [34].

### Studied outcomes

The primary outcome of interest was treatment success in patients that had bacteremia from isolates susceptible to a beta-lactam and at least one of the two other studied antimicrobial categories (aminoglycosides or quinolones) and who received either beta-lactam as monotherapy or in combination with another active antimicrobial. Treatment was considered successful if all signs and symptoms of infection due to *Pseudomonas aeruginosa* were absent after the end of antimicrobial therapy and remained so for at least one week. Any blood cultures after the beginning of therapy and for at least one week after its discontinuation should also be negative for *Pseudomonas aeruginosa*. Therapy was considered as failure if re-isolation of *Pseudomonas aeruginosa* in blood or other tissues occurred within a week after the end of therapy, if the patient had no clinical improvement or had deterioration, or if death due to infection occurred during therapy.

If therapy was changed due to a superinfection with another microbe or it was discontinued due to toxicity, the outcome was evaluated on the basis of the clinical response that the patient had until the moment the regimen was changed if this was straightforward (clearly improving or deteriorating), otherwise the case was excluded from further analysis. Secondary outcomes of interest were: i) treatment success in patients that received the same empirical and definitive regimen, ii) toxicity caused by the antimicrobial agent, requiring discontinuation of it or significant dose adjustment (decrease larger than 1/3 of the original dose, and iii) infection-related and all-cause mortality.

### Microbiological testing

Identification and susceptibility testing of the *P. aeruginosa* isolates were performed by using an automated broth microdilution method (bioMerieux, Vitek II, Hazelwood, MΟ). The in vitro susceptibility of the *P. aeruginosa* blood isolates to amikacin, gentamicin, netilmicin, tobramycin, ciprofloxacin, pefloxacin, aztreonam, ticarcillin, ticarcillin/clavulanic acid, piperacillin, piperacillin/tazobactam, ceftazidime, cefepime, cefpirome, imipenem, meropenem, trimethoprim/sulfamethoxazole, colistin, and other antimicrobials was tested. The breakpoints were those defined by the Clinical and Laboratory Standards Institute (CLSI) [35], [36].

### Statistical analysis

Baseline characteristics of patients that received beta-lactam monotherapy were compared with those of patients that received combination therapy. In addition, the same variables were compared in patients with and without treatment success in order to identify factors associated with success. The rare instances of different episodes in the same patient were analyzed together with the rest of patient-episodes, with the exception of mortality outcomes. Categorical variables were compared by Fischer's exact test and relevant exact odds ratios (ORs) were presented for the main findings. For continuous variables, we used the Student's t-test or the Mann-Whitney test for normally and non-normally distributed variables, respectively. In bivariable analyses of exposure (therapy) and outcome, adjustments were made for all variables (baseline characteristics) that differed significantly (p<0.05) between the monotherapy versus combination therapy groups, evaluating for possible treatment modification (interaction). A multivariable logistic regression analysis of risk factors for treatment success was formed. Variables included were those related to the outcome of interest in bivariable analyses and those unevenly distributed among the two study groups at a level of p-value<0.05. Variables included in the final multivariable model were further examined to exclude any collinearity among them. Type of therapy (combination versus monotherapy) is the only variable that would be included in a separate multivariable analysis, in case of non-significance in bivariable analyses. All statistical analyses were performed using Stata 9.2 statistical software (StataCorp LP, TX, USA).

## Results

Ninety-two patient-episodes with *Pseudomonas aeruginosa* bacteremia were identified in the databases of the included hospitals. A significant proportion of them had multiresistant strains susceptible only to colistin (17 episodes). In 4 episodes *Pseudomonas aeruginosa* was susceptible only to a beta lactam, in 4 episodes susceptible only to an aminoglycoside or quinolone, 4 episodes were treated with ciprofloxacin monotherapy, and 1 episode received 3 antimicrobials from the included classes, thus all these episodes were excluded. Two patients died within 12 hours from admission and in 3 other cases important data regarding therapy and outcome were not included in the medical records. Overall 57 episodes of PA bacteremia (in 53 patients) were treated for sufficient duration with appropriate empiric therapy (39 episodes), definitive therapy (54 episodes), or both (36 episodes). All patients had sepsis, defined by at least two clinical manifestations of SIRS (fever or hypothermia, tachycardia, tachypnea, and/or abnormal WBC count).

### Primary outcome

There were 54 evaluable episodes in 50 patients for the primary outcome of interest, i.e. treatment success in episodes of bacteremia due to isolates susceptible to a beta-lactam and an aminoglyscoside or a quinolone and were treated either by monotherapy or combination therapy. Twenty two episodes occurred in Athens, 17 in Crete, and 15 in Italy. Twenty eight (52%) were in female patients, with median age 56 (25–87) years and median duration of hospitalization prior to bacteremia 4.5 (0–125) days. In Italy all 15 episodes were admitted in an infectious diseases department whereas in Greece 14 (36%) were hospitalized in internal medicine department, 7 (18%) in oncology, 8 (21%) in surgery, 5 (13%) in ICU and the rest in various departments. Ten out of fifteen (66%) episodes in Italy occurred in HIV positive patients, since the National Institute for Infectious Diseases in Italy is a reference centre for HIV.

A total of 20 episodes (37%) received monotherapy and the rest 34 received combination of a beta-lactam with an aminoglycoside or a quinolone [20 (37%) and 14 (26%) episodes, respectively]. The most commonly used beta-lactam as definitive therapy was ceftazidime, piperacillin (plus tazobactam), imipenem (plus cilastatin), and meropenem used in 20 (37%), 15 (28%), 6 (11%), and 6 (11%) episodes of which in 8, 6, 4, and 1 were administered as monotherapy, respectively. Amikacin and ciprofloxacin were the most commonly used aminoglycoside and quinolone, used in 60% and 86% of relevant combinations, respectively. Steroid therapy, blood products transfusions, and bone marrow-stimulating factors were used with similar frequencies in the two treatment groups. In 25 (46%) cases bacteremia was primary whereas a central venous catheter was the source in another 11% of cases. Respiratory tract, urinary tract, and abdomen were the source of infection in 17%, 4%, and 15% of the infections, respectively.

Table 1 shows baseline characteristics of patients that received monotherapy compared to those that received combination therapy. As shown, monotherapy was used more often compared to combination therapy in patients with HIV whereas the opposite was true for those that had been hospitalized in the ICU prior to or at the time of the bacteremia. Regarding the Charlson comorbidity index, a high number of points (six) is assigned to a patient with HIV. Thus, although high Charlson index was observed in patients that received monotherapy compared to combination therapy (5.9 versus 4.1, p = 0.02), this difference was not retained when adjustments for HIV were performed (9.4 versus 7.7, p = 0.2 for patients with HIV and 4 versus 3.7, p = 0.6 for patients without, data not shown). In another comparison (not shown), no significant difference was noted regarding the body source of infection in patients that received monotherapy and those that received combination therapy. Finally, no significant difference existed between treatment groups regarding the presence of organ failure (cardiovascular, respiratory, renal, hepatic, and bone marrow), as shown in Table 1.

**Table 1:** Comparison of baseline characteristics and primary outcome in beta-lactam monotherapy and combination therapy groups.

Definitive therapy	Monotherapy, n = 20	Combination therapy n = 34	*p-value*
(*SE, 95% percentiles, or %*)			
Age	64.5 (31.5–81)	55 (31–81)	*0.94* *
Female	8 (*40*)	20 (*59*)	*0.26*
Location:			*0.16*
- Athens	5 (23)	17 (77)	
- Rome	8 (53)	7 (47)	
- Heraklion	7 (41)	10 (59)	
Days hospitalized prior to bacteremia	3 (0–94)	5 (0–61)	*0.47* *
Patients with very long hospitalization prior to bacteremia	2 (*10*)	2 (6)	*0.62*
Hospitalization in ICU prior to bacteremia	1 (*5*)	12 (*35*)	***0.02***
Bacteremia during ICU stay	1 (*5*)	10 (*29*)	***0.04***
Cardiovascular dysfunction**	2 (*10*)	7 (*21*)	*0.46*
Renal dysfunction**	6 (*30*)	5 (*15*)	*0.29*
Respiratory dysfunction**	4 (*20*)	7 (*21*)	*1*
Hepatic dysfunction**	4 (*20*)	7 (*20*)	*1*
Hematologic dysfunction**	4 (*20*)	6 (*18*)	*1*
Solid tumor	8 (*40*)	13 (*38*)	*1*
Neutropenia†	3 (*15*)	5 (*15*)	*1*
Recent chemotherapy	4 (*20*)	10 (*30*)	*0.53*
HIV	7 (*35*)	3 (*8*)	***0.03***
- AIDS	6 (30)	3 (8)	*0.06*
DM	2 (*10*)	10 (*29*)	*0.17*
Operations prior to isolation	1 (*5*)	8 (*24*)	*0.13*
Charlson comorbidity index	5.9 (±*0.8*)	4.1 (±*0.4*)	***0.02***
Age - adjusted Charlson comorbidity index	7.6 (±*0.6*)	5.5 (±*0.5*)	***0.01***
Primary bacteremia	10 (*50*)	15 (*44*)	*0.78*
Other organism in blood culture	1 (5)	7 (21)	*0.23*
Days of delay in administration of appropriate therapy	0 (0–4.5)	0 (0–3)	*0.93* *

*Not normal distribution, median values presented, p-value calculated by Mann-Whitney test.

**Defined as any degree of *failure* resulting from acute or chronic pathology, not including low CD4.

† Alone or as part of pancytopenia.

Table 2 shows the characteristics of patients that had a successful primary outcome compared to those that had treatment failures. In 42 episodes (77%) treatment was considered successful. Use of combination therapy was found to be associated with higher proportions of treatment success in univariable analysis compared to beta-lactam monotherapy [29/34 (85%) in combination versus 13/20 (65%) in monotherapy], however this did not reach statistical significance [odds ratio (OR) 3.1; 95% Confidence Interval (CI) 0.69–14.7, p = 0.1 for treatment success in patients that received combination therapy over those that received monotherapy]. Separate adjustments for all factors presented in Table 2 did not alter significantly the effect that combination therapy had on the outcome. Finally, a bivariable analysis showed no association between the various sites of infection and treatment success. Sites included respiratory and urinary tract, skin and soft tissues, intravenous catheters (central veins), intra-abodminal and other sites.

**Table 2:** Comparison of baseline characteristics of patients with and without treatment success.

Outcome	Success (n = 42)	Failure (n = 12)	*p-value*
(*SE, 95% percentiles, or %*)			
Age	57 (31–81)	55.5 (32–77)	*0.55* *
Female	22 (*52*)	6 (*50*)	*1*
Hospital:			*0.55*
- Athens	18	4	
- Rome	10	5	
- Heraklion	14	3	
Days hospitalized prior to bacteremia	4.5 (0–57)	4.5 (0–125)	*0.52* *
Patients with very long hospitalization prior to bacteremia	1 (2)	3 (25)	***0.03***
Hospitalization in ICU prior to bacteremia	11 (*26*)	2 (*17*)	*0.71*
Bacteremia during ICU stay	10 (24)	1 (8)	*0.42*
Cardiovascular dysfunction**	8 (*19*)	1 (*8*)	*0.67*
Renal dysfunction**	8 (*19*)	3 (*25*)	*0.69*
Respiratory dysfunction**	7 (*17*)	4 (*33*)	*0.24*
Hepatic dysfunction**	10 (*24*)	1 (*8*)	*0.42*
Hematologic dysfunction**	7 (*17*)	3 (25)	*0.67*
Solid tumor	15 (*36*)	6 (*50*)	*0.50*
Neutropenia†	6 (*14*)	2 (*17*)	*1*
Recent chemotherapy	10 (*24*)	4 (*33*)	*0.49*
HIV	6 (*14*)	4 (*33*)	*0.20*
- AIDS	5 (12)	4 (33)	*0.10*
DM	10 (*24*)	2 (*17*)	*0.71*
Operations prior to isolation	8 (*19*)	1 (*8*)	*0.67*
Charlson comorbidity index	4.5 (±0.4)	5.7 (±0.8)	*0.19*
Age - adjusted Charlson comorbidity index	6.1 (±0.5)	6.9 (±0.7)	*0.39*
Primary bacteremia	20 (*48*)	5 (42)	*0.76*
Other organism in blood culture	7 (17)	1 (8)	*0.67*
Days of delay in administration of appropriate therapy	0 (*0–4*)	0 (*0–5*)	*0.52* *
Combination therapy	29 (*69*)	5 (*42*)	*0.10*
Aminoglycoside combination	17 (*40*)	3 (*25*)	*0.5*

*Not normal distribution, median values presented, p-value calculated by Mann-Whitney test.

**Defined as any degree of *failure* resulting from acute or chronic pathology, not including low CD4.

† Alone or as part of pancytopenia.

As shown, the length of hospitalisation before bacteremia did not have a statistically significant association with treatment success overall. When a cut-off point of 48 hours of hospitalisation prior to bacteremia was set, segregating non-hospital from hospital-acquired infections, again no significant difference in treatment success was identified (81% versus 76% of community versus hospital infections, respectively, had a good outcome, data not shown). On the contrary, the few (n = 4) patients with very long hospitalisations of more than two months had low rates of treatment success (25% versus 82% for the rest of patients, p = 0.03).

Variables with p<0.05 in bivariate analyses presented in Tables 1 and 2 as well as type of therapy (combination versus monotherapy) were included in a logistic regression model (Table 3). As shown, a very long hospitalisation before bacteremia was the only factor that had a statistically significant (negative) association with treatment success in the multivariable model did not substantially alter the results. Elimination of type of therapy and HIV (which is included in Charlson's comorbidity index) did not substantially alter the results. Similarly, when eliminating long hospitalization (since it is relevant to only a small proportion of patients- outliers) treatment success in combination therapy over monotherapy group was: OR 2.7, 95% CI 0.6–12.1, p-value 0.21. Overall, no multivariable model predicted the outcome with more precision than very long hospitalization alone.

**Table 3:** Multivariable analysis of factors possibly associated with treatment success.

Factor	OR	95% Conf. Interval	p-value
Very long (>2 months) hospitalization	0.73	0.01–0.95	***0.046***
Hospitalization in ICU prior to bacteremia	0.67	0.09–4.78	0.69
Age-adjusted Charlson comorbidity index	1.02	0.76–1.38	0.88
HIV	0.59	0.08–4.23	0.60
Combination therapy	3.30	0.63–17.22	0.15

### Secondary outcomes

A subanalysis was performed for patients that received the same empirical and definitive regimen, without any change in treatment after the susceptibility testing results. Appropriate empirical therapy was administered in 39 cases, however 3 were not evaluable for definitive therapy due to resistant isolates (i.e. combination therapy was not an option for definitive therapy). Thus, 36 cases were evaluable both for empirical and definitive therapy. In 12 cases (33%) initial therapy was changed whereas in the rest of cases not. No statistically significant difference in treatment success was noted between the cases in which therapy was changed (8/12) and those that received the same monotherapy (4/6) or combination therapy (14/18) throughout the infection (p = 0.7, Fischer's exact). The group with initial beta-lactam monotherapy did not differ significantly from combination therapy regarding overall treatment success (6/11 versus 20/25 episodes, p = 0.22). Baseline characteristics of patients that are reported in Tables 1 and 2 were also tested for association with appropriate empirical beta-lactam monotherapy or combination therapy in these patients. These characteristics did not differ significantly among the two treatment groups (data not shown).

All-cause mortality did not differ significantly between the two treatment groups [6/31 (19%) versus 8/19 (42%) patients in combination versus monotherapy group, p = 0.11]. None of the factors presented in Table 2 were associated with excess mortality except from CCI and age-adjusted CCI (p = 0.05 and 0.03, respectively). There were 8 deaths due to infection among the 12 failures comprising 15% of all treated patients. Mortality due to infection was 3/31 (10%) versus 5/19 (26%) in combination versus monotherapy group, p = 0.23. Only a very long hospitalization prior to infection was associated with increased infection-related mortality (p = 0.01).

Toxicity was recorded in 4 of 57 episodes that received therapy for sufficient time. In 2 of these episodes aminoglycosides were discontinued from empirical combination therapy due to mild renal toxicity. In another episode, rash possibly associated with the use of piperacillin/tazobactam plus ciprofloxacin in the empirical regimen was treated by intravenous use of antihistaminic drugs without any modification in the antimicrobial therapy administered. Finally. combination of oxacillin added to ceftazidime monotherapy for concomitant infection with *Staphylococcus aureus* resulted in neutropenia and the regimen was changed, however this event is actually non-evaluable for toxicity since it is combination of 2 beta-lactams. Thus, only patients that received combinations of antibiotics had some adverse event, however overall these were few (3/35 versus 0/21 episodes, p = 0.29 by Fischer's exact test).

## Discussion

The results of our study indicate that it is uncertain whether there is benefit in using combinations of beta-lactams with other antibiotics when beta-lactam monotherapy can be administered for *Pseudomonas aeruginosa* bacteremia, according to susceptibility testing results. However, this should be interpreted taking into account that the small population of our study would offer sufficient statistical power to identify large differences between the two treatment groups [37]. Specifically, a minimum of 40% percent difference could be identified with statistical power of approximately 80% if alpha (p value) would be 0.05 (e.g. 90% success for combination versus 50% for monotherapy). Nevertheless, such a large difference did not exist in our study, but instead a 20% difference was noted in favor of combination therapy.

Of interest, our study indicates that treatment success is significantly reduced in patients with very prolonged hospitalization prior to isolation of *Pseudomonas aeruginosa*. Actually, this is a composite risk factor affected by failure to treat the (acute) reason of admission, comorbidities, and their interaction. It should be emphasized that prolonged hospitalization itself differs from chronic comorbidities, a characteristic which was not found to be independently associated with the studied outcome in this study. No significant difference in the primary outcome was noted when the studied cut-off for prior hospitalization was set to 48 hours. Interestingly, *Pseudomonas aeruginosa* bacteraemia within 48 hours after admission in its great majority is actually considered a health-related infection, of patients with recent hospitalisations and manifests as rapidly progressive sepsis in patients presenting from the community [38] [39].

Several interesting results have been published regarding the effect that various therapies have on the outcome of *Pseudomonas aeruginosa* bacteremia. First, inappropriate *empirical* therapy was associated with higher mortality compared to appropriate *empirical* therapy, in a study that reported on 305 *Pseudomonas aeruginosa* bacteremias during a six-year period [40]. In the same study, appropriate *empirical* antimicrobial treatment was more often with combinations of antibiotics (P = 0.01) [40]. On the contrary, Osih et al, did not identify any significant association between appropriate *empirical* therapy and mortality among 167 episodes of bacteremia (OR 0.96, 95% C|I 0.31–2.93) but only a 7% reduction in the mean length of stay in patients with appropriate *empirical* therapy. In our study, outcome of patients was not affected by the choice of initial *empirical* therapy that was continued as the same *definitive* therapy afterwards.

In a study of 100 immunocompetent patients, delayed appropriate *definitive* therapy beyond 52 hours after bacteremia was independently associated with 30-day mortality (OR 4.1; 95% CI 1.2–13.9, P = 0.03) [9]. In our study no such association was found, however we examined it on a day rather than hour-scale. In 127 episodes in a hematologic unit pneumonia, septic shock, neutropenia, delayed and inappropriate *definitive* antibiotic therapy, and unresponsive underlying disease had negative impact on the outcomes [10]. On the other hand, appropriateness of therapy was not associated with better outcomes in a multicenter study that analyzed 148 patients [41]. Nevertheless, according to most reports, in accordance to basic pharmacological principles, one should take for granted that inappropriate *definitive* antibiotic therapy is independently associated with a poor outcome [25], [42]. In our study all episodes received appropriate therapy.

Interestingly, no significant difference in the cure rates was observed with use of *definitive* beta-lactam monotherapy compared to combination therapy, in 230 episodes in cancer patients [43]. However, combination therapy was a small proportion of all episodes with aminoglycoside being administered in 14% of episodes and ciprofloxacin only in 3%. In addition, the definition of therapy as appropriate was not based on susceptibility testing but on the existence of anti-pseudomonal drugs in the initial regimen. In another study, Siegman-Igra et al analysed 57 episodes, in which *definitive* monotherapy versus combination therapy resulted in equal infection-related mortality [25]. Nevertheless, ciprofloxacin monotherapy was also included in the monotherapy group whereas combination group did not necessarily include a beta-lactam [25]. In that study finer measures of outcomes such as treatment success were not reported. Instead, the authors also reported all-cause mortality, which was lower in monotherapy versus combination therapy group [7/42 (17%) versus 7/17 (47%), respectively, p = 0.05].

Finally, in a study of 79 evaluable episodes of *definitive* therapy, Chamot et al found that compared to adequate definitive combination therapy, the risk of death at 30 days was higher with inadequate *definitive* therapy but not with adequate *definitive* monotherapy (adjusted Hazards Ratio 0.70; 95% CI 0.30–1.7) [24]. Our results are in accordance with the aforementioned studies regarding *definitive* therapy, since the difference between combination therapy and monotherapy did not reach statistical significance. Notably, we included only beta-lactam monotherapy cases that, according to the susceptibility testing, could also have received a combination of antibiotics. On the contrary, in the above studies it is not clear how many of the appropriately treated patients in the monotherapy group had isolates that were susceptible only to beta-lactams (including carbapenems), in whom the addition of another agent would not be an option [24], [43]. Thus, all episodes presented in our study had equal chances from a microbiological viewpoint to receive monotherapy or combination therapy although they were not randomized.

Our study has several limitations. First of all, the study population was rather small to detect small differences in outcomes between the two treatment groups [37]. This was mainly due to the narrow inclusion criteria that were used for the two treatment groups based on the susceptibility of *Pseudomonas aeruginosa* isolates. Second, patients from three different hospitals were included, which could lead to heterogeneous populations. However, all patients had infections with isolates of similar susceptibility and received similar health services. The only actual difference was the high proportion of HIV seropositive cases in Italy due to the fact that the hospital is a reference centre for such patients. Of note, this population did not have a different prognosis neither on bivariable nor on multivarible analyses of treatment success. Previous studies have also shown that this population, although younger, has comparable outcome with the seronegative population with *Pseudomonas aeruginosa* bacteremia [42]. Third, an index of severity of illness such as APACHE II would be very useful. However, due to the retrospective design of our study, relevant data would probably be missing for some non-ICU patients or would not be present at the same day of bacteremia. Thus, we presented data regarding failure of vital organs around the time of infection and CCI. Finally, a retrospective study can be more easily subject to forms of bias, especially misclassification of cases. On the other hand, due to a strict methodology no missing values existed in our dataset after the completion of patient data collection.

In conclusion, the results from our study indicate that, in patients with *Pseudomonas aeruginosa* bacteremia, the choice between beta-lactam monotherapy and combination therapy with an aminoglycoside or quinolone does not affect treatment success significantly, or if such difference exists it is not large enough to be identified by the sample size of our study. These results are in agreement with previous studies, which however elaborated different methodology. A multicentre, appropriately designed, randomized study comparing beta-lactam monotherapy with combination therapy for *Pseudomonas aeruginosa* bacteremia if justified.
